# Comparison of Rhizosphere Microbiomes Between Domesticated and Wild Wheat in a Typical Agricultural Field: Insights into Microbial Community Structure and Functional Shifts

**DOI:** 10.3390/jof11030168

**Published:** 2025-02-20

**Authors:** Jie Fang, Mihal Blaschkauer, Assaf Distelfeld, Zihao Liu, Bin Song, Shimon Rachmilevitch, Jonathan M. Adams

**Affiliations:** 1 School of Geography and Ocean Science, Nanjing University, Nanjing 210023, China; fangjie961021@163.com (J.F.);; 2 The Jacob Blaustein Institute for Desert Research, Ben-Gurion University of the Negev, Sede Boker Campus, Be’er Sheva 84990, Israel; miblasch@gmail.com; 3 Department of Evolutionary and Environmental Biology, Faculty of Natural Sciences and the Institute of Evolution, University of Haifa, Haifa 3498838, Israel; 4 College of Bioscience and Biotechnology, Shenyang Agricultural University, Shenyang 110866, China

**Keywords:** domesticated wheat, wild ancestor, rhizosphere microbiome, functional traits, co-occurrence patterns, deterministic processes

## Abstract

While the differences between domesticated crops and their wild relatives have been extensively studied, less is known about their rhizosphere microbiomes, which hold potential for breeding stress-resistant traits. We compared the rhizosphere microbiomes of domesticated wheat (*Triticum aestivum* L.) and its wild ancestor (*Triticum turgidum* ssp. *dicoccoides*) in a typical agricultural field using 16S rRNA and ITS gene sequencing. Our results revealed a high level of conservation in the rhizosphere microbiomes between wild and domesticated wheat, with minimal divergence in community composition and microbial network structure. However, domesticated wheat exhibited a higher prevalence of fungal pathogens and increased functional redundancy, with significant enrichment of genes involved in carbon and nitrogen cycling. The microbial community assemblies in both wheats were predominantly governed by deterministic processes. This suggests that long-term conventional agricultural practices have imposed minor effects on the compositional differences between the microbiomes of wild and domesticated wheat. Nonetheless, the lower abundance of apparent pathogens in the rhizosphere of the wild wheat suggests greater natural biota or innate host plant resistance against pathogenic fungi. This study may provide valuable insights into the host selection, assembly patterns, and functional potential of microbial communities in wild versus domesticated wheat, with implications for manipulating microbial communities in future crop breeding.

## 1. Introduction

Wheat (*Triticum* sp.) occupies over 16% of global cropland and accounts for one-fifth of the food consumed by humans worldwide [1,2]. Ensuring the productivity of global wheat supplies is a major challenge in the face of global climate change, evolving diseases, and environmental damage from intensive farming practices [3,4,5]. Domestication, involving the selection of desirable traits such as fertilizer responsiveness and disease resistance, remains a key strategy for ensuring food security [6], and unintentional interspecific and introgressive hybridizations with wild varieties may contribute to the emergence of subspecies better adapted to agricultural environments [7].

All crops, including wheat, are known to selectively recruit unique microbial communities on their roots, which can co-evolve with the host plant [8,9,10,11]. Previous studies have suggested that the wild ancestors of modern crops harbored microbiomes that were more active and effective in acquiring recalcitrant nutrients, thereby enhancing plant resistance to biotic and abiotic stress [2,12]. In contrast, domesticated crops may have lost the ability to sustain such a microbiome through random mutation. Furthermore, reduced carbon (C) exudation in domesticated crops favored mutant strains that produced higher yields by avoiding the costly investment in what had become an unnecessary microbiome [12,13,14].

Numerous studies have compared the microbiome of wild crop ancestors or traditional cultivars with those of modern high-yielding cultivars [2,15,16,17]. A reduction in microbial species richness has been observed in the rhizosphere microbiome of domesticated crops, accompanied by shifts in taxonomic and functional composition [16]. Modern cultivars typically exhibit a decrease in the relative abundance of Bacteroidota and an increase in Actinomycetota and Pseudomonadota [16]. Nevertheless, contrasting conclusions have been reported, with some studies suggesting that domestication increased the alpha diversity [18] or that only minor differences in rhizosphere microbial composition are associated with host genotype variations [19,20]. Our understanding of how the composition and function of the microbiome in major crops have changed throughout the history of domestication remains incomplete.

On the other hand, previous studies based on pot or plot experiments have provided only limited insight into how domestication processes have influenced the rhizosphere microbiome [16,17,19]. It is essential that studies of the microbiomes of wild crop relatives or ancestors be conducted within the crop’s native region. This approach allows for a realistic understanding of the original microbiome potential of both the crop and its closest wild ancestor, considering the specific climate, soils, and biogeography of the region [18]. Such research could lead to the development of beneficial breeding and microbiome engineering programs aimed at enhancing crop resilience [21,22].

In this study, we selected a wild wheat accession ‘Zavitan’ (*Triticum turgidum* ssp. *dicoccoides*), commonly found as wild emmer [23], and the modern hexaploid wheat cultivar ‘Galil’ (*Triticum aestivum* L.) as our study subjects. Samples were collected from a high-density wheat field in Israel (Aharonson Farm), located within the geographical zone where both modern cultivated wheat and its wild ancestors originated [24,25,26]. This setting provides an ideal context for contrasting the microbiomes of ancestral and modern wheat varieties. We hypothesized that (1) the domestication of wheat and subsequent breeding for commercial traits would have altered the plant’s phenotype, thereby influencing the microbiome they sustain. (2) Specifically, we expected the ancestral wheat to recruit a more diverse and functionally constrained root microbiome, forming more complex and stable communities that were dominated by deterministic processes, which would support the wild plants’ survival under nutrient scarcity, intense abiotic stress, and pathogen attack.

## 2. Materials and Methods

### 2.1. Site Description and Soil Sampling

The wheat field studied was located at the Aharonson Farm for Agricultural Experimentation, near Atlit, Israel (32°41′14″ N, 34°56′18″ E). The field is flat and composed of uniform loam soil, with a pH value of 7.6, organic matter of 8.2%, nitrate nitrogen of 12.3 mg kg^−1^, ammonium nitrogen of 52.9 mg kg^−1^, and available phosphorus of 11.2 mg kg^−1^. The location is characterized by a typical hot summer Mediterranean climate (Csa in the Köppen–Geiger classification), with a mean annual temperature of 20.3 °C and a mean annual precipitation of 560 mm.

Whole plants were randomly harvested from a 50 m^2^ field, when the grain heads of both wild (*Triticum turgidum* ssp. *dicoccoides* var. Zavitan) and domesticated (*Triticum aestivum* L. var. Galil) wheat were still green and swelling. Plants were uprooted by holding the stem and inserting a trowel into the soil about 7 cm away from the stem in a circular motion, carefully lifting the entire root ball (roots and adhering soil) from the ground. The root ball was immediately placed in a sterile bag and kept chilled, arriving at the lab within one hour of sampling. A total of 12 domesticated and 11 wild wheat plants with rhizosphere soils were collected. The plants with roots still attached were vigorously shaken to remove loose soil (outer rhizosphere). The roots were then gently rubbed using sterile gloved fingers (changing to a clean pair of gloves for each plant) to remove the closely attached soil of the middle rhizosphere.

### 2.2. DNA Extraction and Amplicon Sequencing

This middle rhizosphere soil was processed using a Qiagen PowerSoil^®^ DNeasy Isolation Kit for DNA extraction. Samples were sequenced at Dalhouise University Integrated Microbiome Resource. Amplicon fragments were PCR-amplified from the DNA extracted above in duplicates using separate template dilutions (1:1 and 1:10) using high-fidelity Phusion+ polymerase. A single round of PCR was carried out using “fusion primers” (Illumina adaptors + indices + specific regions) targeting the V4-V5 sub-regions of the 16S rRNA (bacteria) and ITS2 (fungi) genes. PacBio Sequel 2 was performed as above, except full-length 16S/ITS fusion primers (PacBio adaptors + barcodes + specific regions) were used instead. PCR products were verified visually by running on a high-throughput Hamilton Nimbus Select robot using Coastal Genomics Analytical Gels. The PCR reactions from the same samples were pooled in one plate and then cleaned-up and normalized using a high-throughput Charm Biotech Just-a-Plate 96-well Normalization Kit. The primers used were as follows: 515F—GTGYCAGCMGCCGCGGTAA—926R—CCGYCAATTYMTTTRAGTTT for bacteria and TS86F—GTGAATCATCGAATCTTTGAA—ITS4R—TCCTCCGCTTATTGATATGC for fungi [27,28]. Due to high plant plasmid and mitochondrial DNA contamination, PNA blockers were added to the PCR stage (mPNA—GGCAAGTGTTCGGA for mitochondrial DNA, pPNA—GGCTCAACCCTGGACAG for plastid), as described in [29]. However, 16S rRNA and ITS gene fragments were successfully amplified from the rhizosphere soil of only 8 pairs of domesticated and wild wheat (i.e., 8 domesticated wheat samples and 8 wild wheat samples), and thus, these 8 pairs were used for subsequent analyses. The raw sequences were submitted to the NCBI Sequence Read Archive (SRA) with accession number PRJNA917337.

### 2.3. Bioinformatic Processing

Amplicon sequences were processed and analyzed with QIIME 2 v.2020.8 (https://qiime2.org; accessed on 14 November 2023), with quality control and actual sequence variants (ASVs) identification performed with the DADA2 pipeline [30]. An ASV table was then created and normalized according to the lowest total number of sequences in the sample to avoid the effect of uneven sequencing depth between samples [31]. Taxonomic assignment of ASVs was performed using a machine learning classifier through the classify-sklearn method in Qiime 2 [32]. The 16S rRNA gene sequences were aligned against the SILVA 138 reference database (https://www.arb-silva.de; accessed on 18 November 2023), while the ITS gene sequences were aligned against UNITE general FASTA release for Fungi version 6.0 (https://unite.ut.ee; accessed on 18 November 2023). Because this study’s focus was limited to investigating the bacterial and fungal communities, archaeal sequences were removed from the 16S rRNA sequences for the subsequent analysis.

### 2.4. Statistical Analysis

#### 2.4.1. Alpha and Beta Diversity

Alpha indices, including the Chao 1, richness, Simpson, and Shannon indices, were calculated based on the normalized ASV table using the “Vegan” package in R 4.1.0 [33]. Differences in alpha diversity indices were tested by the Wilcoxon test.

To demonstrate the bacterial and fungal communities’ differences between the domesticated and wild wheat rhizosphere soils, non-metric multidimensional scaling ordination (NMDS) analysis based on the Bray–Curtis dissimilarity was conducted. Permutational multivariate analysis of variance (PERMANOVA) was used to evaluate the significant differences between the domesticated and wild groups. NMDS and PERMANOVA were conducted using the “phyloseq” and “Vegan” packages in R 4.1.0, respectively [33,34].

#### 2.4.2. Function Prediction

To investigate the potential functions of bacteria, the inferred function from soil bacterial taxonomy was evaluated based on the Functional Annotation of Prokaryotic Taxa database (FAPROTAX) [35]. The top functional groups with the average proportions of bacterial ASVs among all the samples higher than 0.1% were visualized. In addition, the Tax4Fun2 package [36] was used for the prediction of functional redundancy in the microbial community through 16S rRNA gene sequence similarity. Furthermore, the proportion of genes associated with soil C, nitrogen (N), and phosphorus (P) cycling functions based on 16S rRNA gene data was obtained, with 101, 59, and 35 genes related to soil C, N, and P cycling, respectively, being found in soils according to previous studies [37,38].

The investigation of fungal community function was performed with FUNGuild v1.0 [39], which can taxonomically parse fungal ASVs by analyzing the ecological guild of sequencing databases. Three confidence ranks, namely, “highly probable”, “probable”, and “possible”, could be evaluated according to comparison in the fungal database, suggesting the possibility of assumed guilds. The guilds that were “highly probable” and “probable” in the assignments were selected for the purpose of not over-interpreting their data ecologically. All ASVs that did not match taxa in the database were classified as “unassigned”. Then, top functional groups with the average proportions of fungal ASVs among all samples higher than 0.1% were visualized.

#### 2.4.3. Network Analysis

Species co-occurrence networks were constructed for the domesticated and wild wheat rhizospheres, respectively, to illustrate the ecological interactions in their microbial communities. To reduce the complexity of the datasets to facilitate analysis, we constructed single networks for bacteria and fungi only using the most abundant ASVs (top 300 ASVs), respectively, and constructed a joint interdomain network combining the top 300 bacterial and fungal ASVs [40].

Pairwise correlation was calculated based on the SparCC method with default parameters, which was designed to assess correlations for compositional data and construct a non-random network [41]. Robust associations in the matrix were selected under the threshold value of 0.6 and significance of 0.01 [42]. Network visualization was generated with Gephi version 0.9.1 (https://gephi.org; accessed on 20 November 2023), and the layout was generated using the Fruchterman–Reingold force-based algorithm [43]. Each node indicates a given ASV, and each edge represents a significant correlation between two ASVs. Topological parameters were calculated in R version 4.1.0 with the ‘igraph’ package [44]. Furthermore, network parameter values were assigned to each sample to estimate the complexity of the soil network (Supplementary Table S1), and the differences were tested by the Wilcoxon test, including the number of nodes and edges, average degree, and modularity [45,46]. According to the definition of these parameters, higher numbers of nodes and edges, a higher value of average degree, and a lower value of modularity suggest a more connected network, reflecting more potential complexity of soil networks [46,47].

The topological roles of nodes in each species network were identified based on their within-module connectivity (Zi) and among-module connectivity (Pi), which were calculated using the method described in [48,49]. Nodes in the network can be classified as network hubs (Zi > 2.5 and Pi > 0.62), module hubs (Zi > 2.5 and Pi < 0.62), connectors (Zi < 2.5 and Pi > 0.62), and peripherals (Zi < 2.5 and Pi < 0.62). Here, we defined the network hubs, module hubs, and connectors as keystone species.

To further evaluate the species network features, robustness was calculated based on the network natural connectivity, and the network stability was evaluated by removing edges randomly and repetitively to estimate how quickly the network robustness degraded [50,51]. Robustness analysis was shown as linear regression curves fitted by the natural connectivity and the proportion of removed edges, and the slopes of the curves were then compared by performing 1000 permutations using the “Simba” R package [52]. A greater absolute value of slope indicates a weaker network stability [53].

#### 2.4.4. Community Assembly Process

To determine the relative importance of stochastic and deterministic processes for bacterial and fungal communities, phylogenetic normalized stochasticity ratio (pNST) analysis was applied, with pNST < 0.5 indicating a more deterministic assembly [54]. Differences in the pNST index between the domesticated and wild wheat rhizospheres were tested using the Wilcoxon test. A neutral community model (NCM) was used to further assess the potential importance of stochasticity in assembling for both bacterial and fungal communities, as described in Chen et al. [55]. In this model, the parameter “m” assesses the estimated migration rate, and “R^2^” represents the overall fit to the neutral model. The ASVs were separated into three partitions depending on whether they occurred above, below, or within (neutral) the 95% confidence interval of the NCM predictions. All calculations were performed in R v4.1.0, as previously described Chen et al. [55].

## 3. Results

### 3.1. Microbial Alpha Diversity and Community Composition

We found no significant differences in alpha diversity between the rhizosphere microbiomes of the domesticated and wild wheat. Specifically, there were no significant differences in the richness, Chao1, Shannon or Simpson indices between the wild and domesticated wheat in both the bacterial and fungal communities (Supplementary Figure S1).

In both the wild and domesticated wheat, the bacterial community was dominated by Pseudomonadota, with an average relative abundance of 39.25% in the wild and 34.06% in the domesticated wheat rhizospheres, respectively. This was followed by Bacteroidota (29.23% and 20.71%, respectively), Acidobacteriota (11.03% and 11.55%, respectively), and Actinomycetota (9.84% and 8.98%, respectively) (Supplementary Figure S2A). Pseudomonadota were significantly more abundant in the wild wheat rhizosphere, while Bacteroidota were more abundant in domesticated wheat. The relative abundances of other dominant bacterial phyla showed no significant differences between the two rhizospheres.

**Figure 1:**
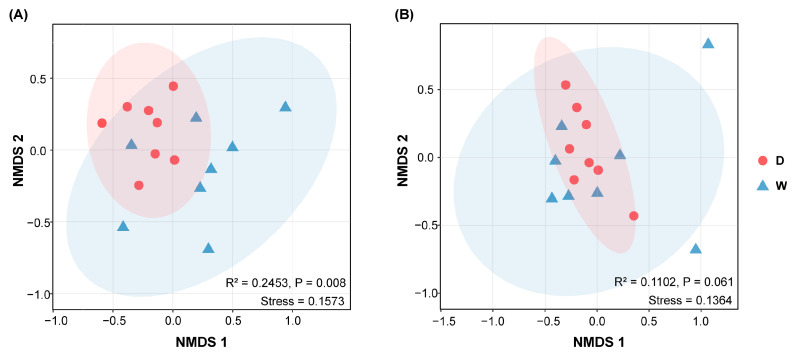
Non-metric multidimensional scaling (NMDS) plots comparing the (**A**) bacterial and (**B**) fungal communities in wild (“W”) and domesticated (“D”) wheat rhizosphere soils based on the pairwise Bray–Curtis distance.

In the fungal community (Supplementary Figure S2B), Ascomycota was the most abundant phylum, with an average relative abundance of 57.68% in the domesticated and 49.81% in the wild wheat rhizosphere. This was followed by Basidiomycota (38.16% and 45.24%, respectively), Chytridiomycota (2.07% and 1.51%, respectively), Zygomycota (0.71% and 0.90%, respectively), and Glomeromycota (0.23% and 0.42%, respectively). No significant differences were observed in the overall fungal community composition between the domesticated and wild wheat rhizospheres. The NMDS plots (Figure 1) further illustrate the minor differences in the bacterial (R^2^ = 0.25, *p* < 0.01) and fungal (R^2^ = 0.11, *p* > 0.05) community structures between the rhizosphere biota of the domesticated and wild wheat at the ASV level.

### 3.2. Predicted Functions of the Microbial Communities

**Figure 2:**
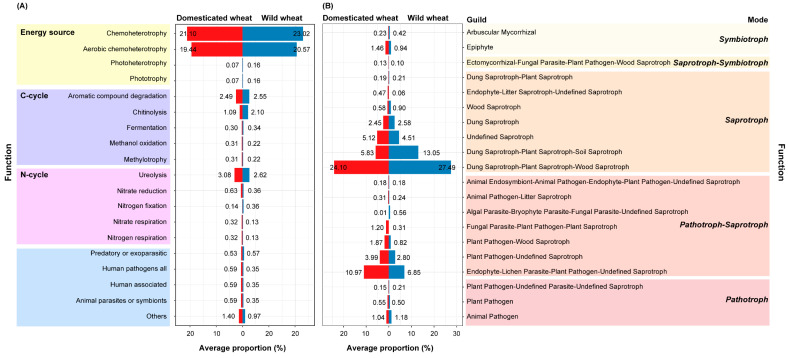
(**A**) Predicted functional profiles of the bacterial communities. Only the inferred functional groups with average proportions of ASVs greater than 0.1% are shown in the plot. (**B**) Predicted functional profiles of the fungal communities. Only the inferred functional groups with a high confidence (indicated as “highly probable” or “probable”) and with average proportions of ASVs higher than 0.1% are shown in the plot.

The potential function predicted by FAPROTAX analysis (Figure 2A) showed that in the bacterial community, chemoheterotrophy (especially aerobic chemoheterotrophy) was the most abundant, followed by ureolysis, aromatic compound degradation, and chitinolysis, whose average proportions were higher than 1%. Except for the function of chitinolysis, which accounted for a relatively higher proportion in the wild wheat rhizosphere, no significant difference was observed in other functions between the wild and domesticated wheats.

**Figure 3:**
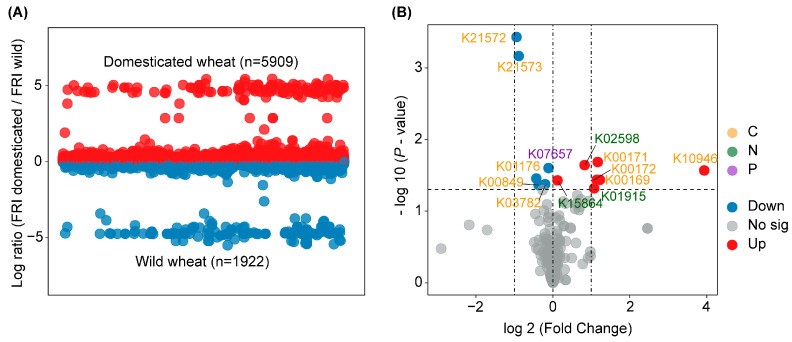
(**A**) Relative functional redundancy indices (FRI) in the rhizosphere of domesticated (red) and wild (blue) wheats, respectively. All predictions were implemented using a 97% similarity cut-off. A log ratio greater than 0 indicates that a function was more redundant in the domesticated wheat. (**B**) Volcano plot showing the difference in the relative abundance of functional genes involved in C, N, and P cycling between the rhizosphere of domesticated (red) and wild (blue) wheat.

The FRI was applied to compare functional redundancy between the rhizosphere bacteria of the domesticated and wild wheat, revealing higher functional redundancy in the domesticated wheat than in the wild wheat (Figure 3A). Approximately 5909 functions from bacteria displayed higher functional redundancies in the domesticated rhizosphere samples, while only 1922 functions showed higher redundancy in the wild groups. The volcano plot indicated that wheat domestication significantly altered the functional genes in the rhizosphere soils (Figure 3B). For instance, the relative abundance of genes involved in the soil C cycle (e.g., K00171, K00172, K00169, and K10946) and N cycle (K01915) were enriched in the domesticated wheat soils.

For the fungal community, the results based on the FUNGuild database identified five trophic modes assigned to the samples (Figure 2B). The most abundant guild was Dung Saprotroph-Plant Saprotroph-Wood Saprotroph, followed by Dung Saprotroph-Plant Saprotroph-Soil Saprotroph and Endophyte-Lichen Parasite-Plant Pathogen-Undefined Saprotroph, for both wild and domesticated wheat. Among them, Endophyte-Lichen Parasite-Plant Pathogen-Undefined Saprotroph, Fungal Parasite Plant Pathogen Plant Saprotroph, Endophyte Litter Saprotroph Undefined Saprotroph, and Plant Pathogen-Wood Saprotroph accounted for a significantly higher proportion in the domesticated wheat than in the wild wheat (*p* < 0.05, tested by the Wilcoxon test).

### 3.3. Co-Occurrence Networks of Soil Microorganisms

**Figure 4:**
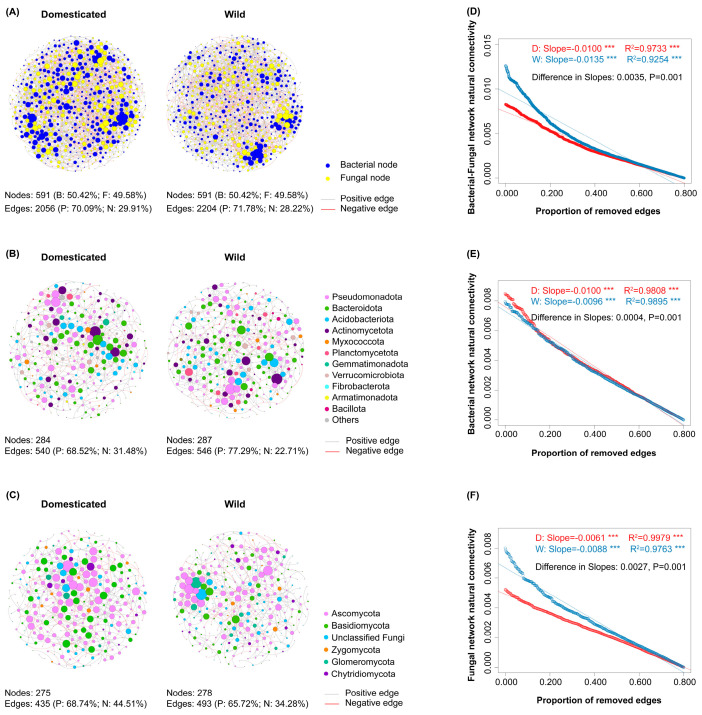
Networks and the stability of the network structures for microbial communities in rhizosphere soils of domesticated and wild wheat. (**A**) Bacteria–fungi networks based on top 300 abundant bacterial and fungal ASVs, (**B**) bacteria-bacteria network based on top 300 abundant bacterial ASVs, and (**C**) fungi-fungi network based on top 300 abundant fungal ASVs selected from rhizosphere soil of domesticated and wild wheat. A node represents a microbial ASV, with the size of each node being proportional to the degree and the color of each node representing bacterial or fungal phylum. An edge represents a strong (|r| > 0.6) and significant (*p* < 0.01) correlation between paired ASVs. (**D**) Stability for bacteria–fungi joint network, (**E**) bacteria-bacteria network, and (**F**) fungi-fungi networks. ***, *p* < 0.001 “W” represents wild and “D” represents domesticated wheat network.

In the bacteria–fungi networks, there were 591 nodes and 2056 edges in the domesticated wheat rhizosphere, compared to 591 nodes and 2204 edges in the wild wheat rhizosphere (Figure 4A). In the bacteria-bacteria networks, there were 284 nodes and 540 edges in the domesticated wheat rhizosphere, while the wild wheat rhizosphere had 287 nodes and 546 edges. The nodes in these networks were primarily affiliated with Pseudomonadota (Figure 4B). For the fungi-fungi networks, there were 275 nodes and 435 edges in the networks of the domesticated wheat rhizospheres, while there were 278 nodes and 493 edges in the wild wheat rhizospheres, with most of these nodes affiliated to Ascomycota (Figure 4C).

Among all these networks, positive edges were more commonly observed than negative ones, indicating that positive cooperation predominantly characterized the connections (Figure 4). Additionally, in the bacteria–fungi joint networks and the fungi-fungi networks, wild wheat rhizospheres had a significantly higher proportion of positive edges when compared to domesticated wheat rhizospheres (Supplementary Figure S3). However, except for the number of nodes and the proportions of negative/positive edges, there were no significant differences in the topological structure of the microbial networks for the bacterial and fungal communities in the domesticated and wild wheat rhizospheres.

We further tested the stability of the microbial networks by assessing the slopes of decreased natural connectivity with the removal of edges. This showed that the natural connectivity of both the joint and fungi-fungi networks decreased to a greater degree in the wild than in the domesticated wheat rhizospheres when the same proportion of edges were removed (Figure 4D–F), indicating greater network stability in the domesticated wheat, while the bacteria-bacteria networks were the opposite.

### 3.4. Keystone Species in Microbial Networks

For both the joint bacteria–fungi and fungi-only networks, there were more keystones present in the domesticated wheat rhizosphere (Supplementary Figure S4). In the bacteria–fungi networks, nearly half of the nodes were fungal ASVs (among 591 nodes in both the domesticated and wild wheat, a total of 293 nodes were fungal ASVs). However, only 8 and 13 fungal ASVs were keystones, with all of them functioning as connectors (Supplementary Table S2).

The role assumed by individual ASVs in the networks rarely overlapped between the domesticated and wild wheat rhizospheres. Only a few ASVs acted as keystones in the networks of both types of wheat. For example, in the joint bacteria–fungi networks, only two bacterial ASVs (B_ASV43 *Dyadobacter* and B_ASV55 unclassified *Comamonadaceae*) maintained their roles as connectors in both wheat types (Supplementary Table S2). Additionally, one bacterial module hub in the domesticated wheat rhizosphere (B_ASV17, unclassified *Chitinophagaceae*) transitioned to a connector role in the wild wheat rhizosphere. In the bacteria-only networks, the module hub B_ASV14 (*Altererythrobacter*) in the domesticated wheat rhizosphere shifted to being a connector in the wild wheat rhizosphere (Supplementary Table S3).

Only a small proportion of the keystone species in the community can be affiliated to any predicted ecological function (Supplementary Tables S2–S4). Among these keystone species, most of the bacteria were inferred to be related to aerobic chemoheterotrophy, while most of the fungi were saprotrophs (Supplementary Tables S2–S4). In addition, some bacteria (B_ASV110 and B_ASV41, for instance) involved in N cycling acted as connectors in the joint networks for the wild wheat rhizosphere.

### 3.5. Differences in the Microbial Assembly Processes in the Rhizosphere Soils of Wild and Domesticated Wheats

**Figure 5:**
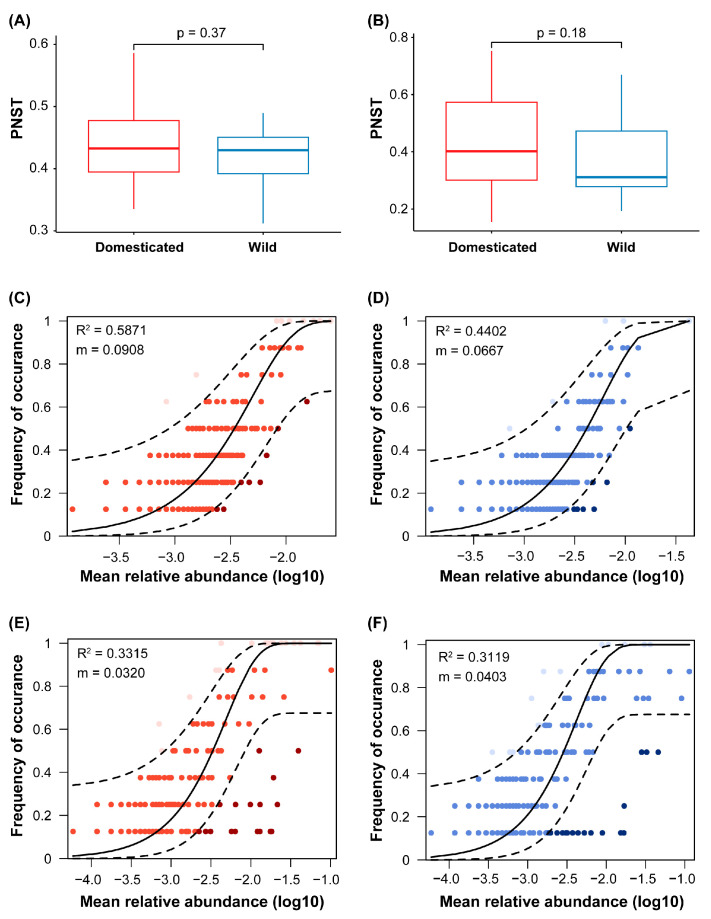
Phylogenetic normalized stochasticity ratio (pNST) for (**A**) bacterial and (**B**) fungal community in domesticated and wild wheat soils. The differences were compared using pairwise Wilcoxon tests. Neutral community model (NCM) plots for (**C**,**D**) bacterial and (**E**,**F**) fungal communities. Dashed lines indicate the 95% confidence intervals around all fitting statistics based on bootstrapping with 1000 bootstrap replicates. Points shown between the two dashed lines represent the operational taxonomic units (ASVs) with the frequency of occurrence within the 95% confidence intervals around the NCM prediction. ASVs that occurred more frequently than the value predicted by the model are shown in a lighter color, while those that occurred less frequently than predicted are shown in a darker color. R^2^ represents the overall fit to the neutral model. The parameter “m” assesses the estimated migration rate.

For the rhizosphere of both the wild and domesticated wheat, the pNST index revealed the dominance of a deterministic process in structuring both the bacterial and fungal communities (Figure 5A,B), with the median of the pNST value being less than 0.5, but there was no significant difference between the domesticated and wild wheats. The goodness-of-fit to the neutral model of the fungal communities was lower than that of the bacterial communities, and it was lower in the microbial communities of the wild wheat than of the domesticated wheat (Figure 5C–F).

## 4. Discussion

### 4.1. Similar Microbial Community Diversity of the Wild and Domesticated Wheat

We hypothesized that wheat domestication and subsequent breeding for commercial traits would have changed the microbiome composition, with the domesticated wheat ancestor harboring a less diverse rhizosphere microbiome. However, the comparison showed no significant difference in alpha diversity between the wild and domesticated wheat (Supplementary Figure S1). Furthermore, a significant difference was only observed in the bacterial community structure (Figure 1), while the relative abundance of fungal phyla did not differ significantly between the wild and domesticated wheat (Supplementary Figure S2B). The history of domestication in nutrient-enriched agricultural environments might be expected to have selected against heavy investment by the plant in AMF fungi. For example, the ancient Glomeromycota families (e.g., Archaeosporaceae and Paraglomeraceae) were thought to be less beneficial for plant growth and nutrient uptake than more recent families (e.g., Glomeraceae) [56]. Here, there were no significant differences in either the relative abundance of Glomeromycota (Supplementary Figure S2B) or the symbiotroph guild predicted by FUNGuild (Figure 2B) between the wild and domesticated wheat. Similar results were also observed for some mutualistic bacterial groups, such as Rhizobium/Bradyrhizobium, further confirming the conserved nature of wheat microbiota despite the influence of domestication and hybridization.

The lack of differences in the rhizosphere microbial communities between the wild and domesticated species in this study may not only be due to differences in root secretions but also due to the growth stage of the plant, which can significantly impact the composition and diversity of the microbiome [16,57,58]. As shown by Gholizadeh et al. [58], the diversity and structure of the rhizosphere microbial community of wild and domesticated species differed more strikingly in early stages of growth than in later stages. The high-input field provided a favorable environment for microbial survival, enabling rapid colonization [59]. By the time of sampling, the C allocation would potentially have shifted from the belowground (i.e., roots/microbiome) to the developing wheat grains, resulting in the stabilization of the rhizosphere microbial community composition [16,58]. Furthermore, the presence of common microbiome members in the rhizosphere (e.g., Pseudomonadota, Bacteroidota, Actinomycetota, and Ascomycota) has been consistently observed in wheat and other crops, indicating the potential for the existence of a highly conserved, coevolved, and host-independent core microbial taxa that plays a role in facilitating microbial cooperation and maintaining crop fitness [60,61].

### 4.2. Functional Differences Between the Domesticated and Wild Wheat Rhizobiome

We hypothesized that the wild wheat would have a functionally integrated root microbiome in order to support a range of functions necessary for survival. The results showed that the predicted functions of the bacterial communities in the rhizosphere of the two wheats exhibited negligible compositional divergence (Figure 2A). However, the higher FRI values indicated that the domesticated rhizosphere bacteria had a greater number of redundant functions than the wild wheat (Figure 3A) [36]. High functional redundancy is crucial for maintaining ecological processes facing disturbance or species extinction, especially in maintaining ecosystem productivity, C sequestration, and nutrient cycling, which play an essential role in enhancing ecosystem resilience and stability [62,63]. Combining with the enriched C cycling genes in the domesticated wheat rhizosphere (Figure 3B), these results suggest that the bacterial members of the domesticated wheat rhizosphere soils underwent functional changes and adapted well to the microenvironment or substrate renewal in the habitat and to local agricultural practices [64].

Meanwhile, our results showed that domestication and associated agricultural practices may have reduced the ability of the domesticated wheat rhizosphere microbiota to naturally control fungal pathogens, since the relative abundance of inferred plant pathogenic fungi was higher among the fungal communities in the rhizosphere of the domesticated wheat than in the wild wheat (Figure 2B). Root exudates are important in shaping the rhizosphere the microbial community, with several compounds found in root exudates that may recruit beneficial bacteria or have an effect on the expression of bacterial antifungal biosynthetic genes, presumably as a plant systemic response to deal with pathogens [13]. According to Kapulnik and Kushnir [65] and Valente et al. [66], wild ancient wheat varieties were more capable of interacting with beneficial plant-growth-promoting rhizobacteria. The advancement of conventional agricultural techniques and alterations in plant physiology and morphology throughout the process of domestication have also shaped the environmental guilds of the rhizosphere-associated fungal microbiome [67]. Protection by pesticides may make it unnecessary for the root systems of modern cultivars to continuously maintain this ability to recruit beneficial bacteria to resist pests and diseases, and frustratingly, it may have reduced crop resistance to potential biotic stresses [2]. As it is difficult to determine whether fungi are symbiotic or pathogenic merely based on taxonomy, these patterns need to be further investigated in future studies and inspire future breeding efforts to use rhizosphere microbial traits as indicators of crop development [20,39].

### 4.3. Similar Cooccurrence Network Pattern of Domesticated and Wild Wheat Rhizobiome

Despite its limitations, network analysis can help us understand the significance of rhizosphere microbes for plant growth [14]. We hypothesized that there would be greater network complexity and stability in the microbial community of the wild wheat rhizosphere compared to the domesticated wheat. However, our results showed that there was no significant difference in topological features except for fewer positive connections in the joint bacteria–fungi network and fungi-only network of the domesticated wheat than those of the wild wheat (Figure 4, Supplementary Figure S3). In addition, the joint bacteria–fungi network and fungi-only network of the domesticated wheat were more stable (Figure 4).

Despite the fact that some studies on soybean and rice [18,68] have previously found that wild ancestors of crop plants have more complex and tighter associations amongst the community of root-associated microorganisms, the extent to which rhizosphere microorganisms develop deleterious, beneficial, or neutral interactions with each other, and the density and complexity of the resulting interaction networks, depends on taxa richness, microbial abundance, and activity levels [11,69,70,71]. As mentioned above, the discrepancy in the diversity and composition of the rhizosphere microbes was negligible, and the microbial interaction network structures were inevitably similar.

One interesting difference, however, is the reduced proportion of positive connections in the joint bacteria–fungi network and the fungi-only network for the domesticated wheat (Supplementary Figure S3). Considering that crop domestication is a process driven by loss-of-function alleles and indels in plant genomes, domesticated wheat might have lost the genes responsible for mutualistic relationships with fungal species [72]. The ratio of negative associations to positive associations between taxa can be used as a property to predict the stability of co-occurrence networks, with positive relationships representing high niche overlap and negative relationships indicating divergent niches [73]. Positive interactions can destabilize microbial communities by creating positive feedback loops between taxa supporting each other’s fitness [73]. Our results showing that the joint bacteria–fungi network and fungi-only network of the domesticated wheat with a smaller proportion of positive edges were more stable than those of the wild wheat also correspond to this viewpoint.

Our results showed that the domesticated wheat rhizo-biome harbored more keystone species than the networks of the wild wheat (Supplementary Figure S4). Keystone species are usually highly connected with other species within networks and potentially have a considerable influence on the entire microbial community [74,75]. Our results aligned with previous research suggesting that agricultural practice might affect the abundance of keystone taxa in rhizo-microbiome networks [75].

We also found that the representative ASVs for each keystone category in the domesticated and wild wheat networks were rarely the same. Each identifiable type of microorganism has multiple biogeochemical functions, and each function is often shared by different strains and species, leading to functional redundancy in microbial communities [76]. For root-associated microorganisms, a variety of microorganisms may maintain biogeochemical processes by assuming various functional roles [77,78,79]. In addition, although taxonomic differences within individual functional groups do not appear to fluctuate significantly with soil and other environmental conditions, the functional potential of microbiota is thought to be closely related to environmental conditions (soil physico-chemical properties, plant genotype, and growth stage) [76,78,80]. Therefore, similar environments should have promoted similar microbial communities while allowing for taxonomic differences within individual functional groups.

### 4.4. Deterministic Processes Dominate Microbial Community Assembly for Both Domesticated and Wild Wheat Rhizosphere Soils

We had hypothesized that wild wheat would have a more functionally selected deterministic community structure in its rhizosphere. However, our analysis revealed that deterministic processes dominated community assemblies for both domesticated and wild wheat rhizobiomes (Figure 5), a finding which is consistent with studies on agricultural soils for domesticated wheat in northern [81] and eastern China [82,83].

It is important, however, to note that neither the wild nor the domesticated wheat in this study were growing in a soil that truly resembled the preagricultural state. It is known that rhizobiomes are influenced by interactions between plant genotype, soil, and environmental stresses [20,84], and in this study, the wild and domesticated plants were growing in a high-density wheat field in close proximity. In this wheat field setting, where they grew intermingled, both types of wheat would thus have experienced the same conditions, both abiotic and biotic, for example, enhanced water availability in deeper and well-conditioned soils, increased nutrient input from chemical fertilizer, and spraying with pesticides. According to the habitat and environmental filtering perspectives [81,82,83], a one-to-one match may exist between niche availability and community structure, which could explain the similarity in microbiomes between the domesticated and wild wheats. It is also important to note that the wild wheat population here has likely grown for many generations as a weed in a wheat field under high-intensity modern farming—a situation very different from those in which its own wild ancestors lived. The wild wheat population has been self-sowing and subject to essentially the same wheat field environment as domesticated wheat. In this sense, both the wild and domesticated wheats have likely experienced evolutionary selection under the high-nutrient and pesticide-protected conditions of modern intensive farming.

Nevertheless, despite the overall similarity, we did find a somewhat more prominent role of neutral processes in the assembly of the microbiota of domesticated wheat, and we propose that domestication may have relaxed selective processes in the assembly of the wheat microbiota. This finding suggests that, as expected, wild wheat does exert more selection and control on the composition of its rhizosphere microbiota [79]. In addition, future studies should examine the microbiomes of other plants (e.g., weeds) in the same field to gain a deeper insight into whether it is the plant type/genotype, soil characteristics, or other environmental stresses that drive the conserved microbiome structure.

## 5. Conclusions

Given the various studies demonstrating differences in root-associated microbiome between domesticated crops and their relatives, the findings from our comparison of two related wheats growing in the same field was surprising. In this context, the wheat rhizobiome appears to be highly conserved in community composition, despite the two lineages having been separate for around 10,000 years of breeding and selection by the agricultural environment, as well as having incorporated other genomes (e.g., *Aegilops*) in hybridization events [85]. This lack of difference may be attributed to the fact that both the domesticated and the wild wheat grew in the same fields and were subject to similar selection pressures regarding their ability to support or suppress components of their microbiome. However, since the wild wheat itself is presumably well adapted to the crop field environment, its features may be more relevant to breeders. It remains possible that differences do exist at the root surface or within the root (the endosphere), but there are no strong indications of such differences in the rhizosphere. Meanwhile, our results revealed that the rhizosphere of domesticated wheat displayed higher functional redundancy, with a significant enrichment of genes involved in carbon and nitrogen cycling. Furthermore, a greater prevalence of inferred fungal pathogens was observed in the rhizosphere of domesticated wheat compared to that of wild wheat. This shift was likely due to hybridization and selective breeding during domestication, which may have raised a functional shift in the rhizosphere microbiome to help increase the crop’s resource utilization efficiency while reducing the crop’s native resistance to potential pathogens.

This study has deepened our understanding of the rhizosphere microorganisms on wheat by comparing domesticated wheat and its wild relative directly in a naturally agricultural field environment. It appears, from this study at least, that we should regard rhizosphere microbial traits as an indicator for the development of crops in future breeding work.

## Data Availability

The obtained sequences were submitted to the NCBI Sequence Read Archive (SRA) with accession number PRJNA917337.

## Supplementary Materials

The following supporting information can be downloaded at: https://www.mdpi.com/article/10.3390/jof11030168/s1. Figure S1: Alpha diversity including Chao 1, Richness, Simpson and Shannon indexes for bacterial (A) and fungal (B) communities; Figure S2: Boxplots comparing the relative abundances of most abundant bacterial (A) and fungal (B) phylum in rhizosphere soils of the wild wheat and domesticated wheat; Figure S3: Microbial network topologic feature comparison between domesticated and wild wheat; Figure S4: Keystone species in microbial networks. Table S1: Definitions of topological parameters; Table S2: Information of keystones in the bacteria-fungi joint networks in domesticated and wild wheat soils; Table S3: Information of keystones in the bacteria single networks in domesticated and wild wheat soils. Table S4: Information of keystones in the fungi single networks in domesticated and wild wheat soils.
