# Supplementary material for: Comparison of Rhizosphere Microbiomes Between Domesticated and Wild Wheat in a Typical Agricultural Field: Insights into Microbial Community Structure and Functional Shifts
Source: J Fungi (Basel). 2025 Feb 20;11(3):168. doi: 10.3390/jof11030168 (PMC11943456; doi:10.3390/jof11030168)
Supplement: Supplementary file 1 [file jof-11-00168-s001.zip › Supplementary Figures.pdf]

Figure list:

Supplementary Figure S1

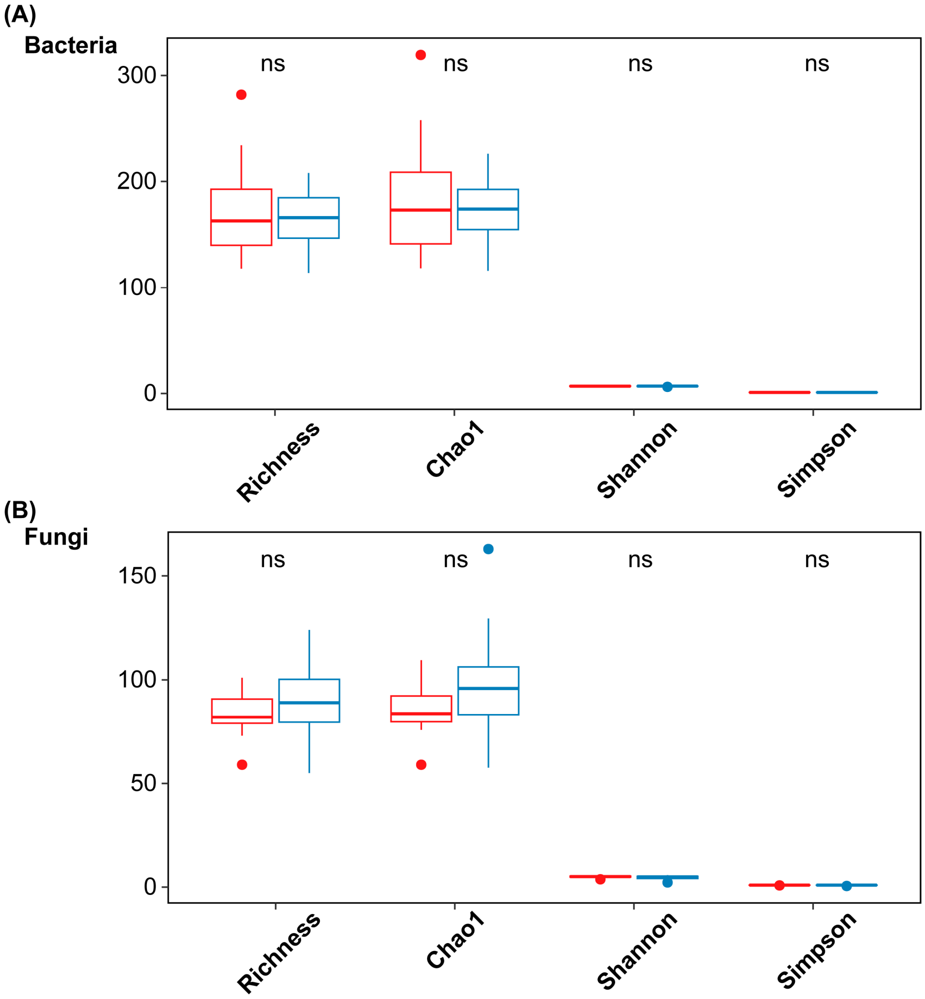

Supplementary Figure S1 Alpha diversity including Chao 1, Richness, Simpson and Shannon indexes for bacterial (A) and fungal (B) communities. Statistical differences were analyzed using a pairwise Wilcoxon test, *** $p < 0.001$, ** $p < 0.01$, * $p < 0.05$; ns, not significant

Supplementary Figure S2

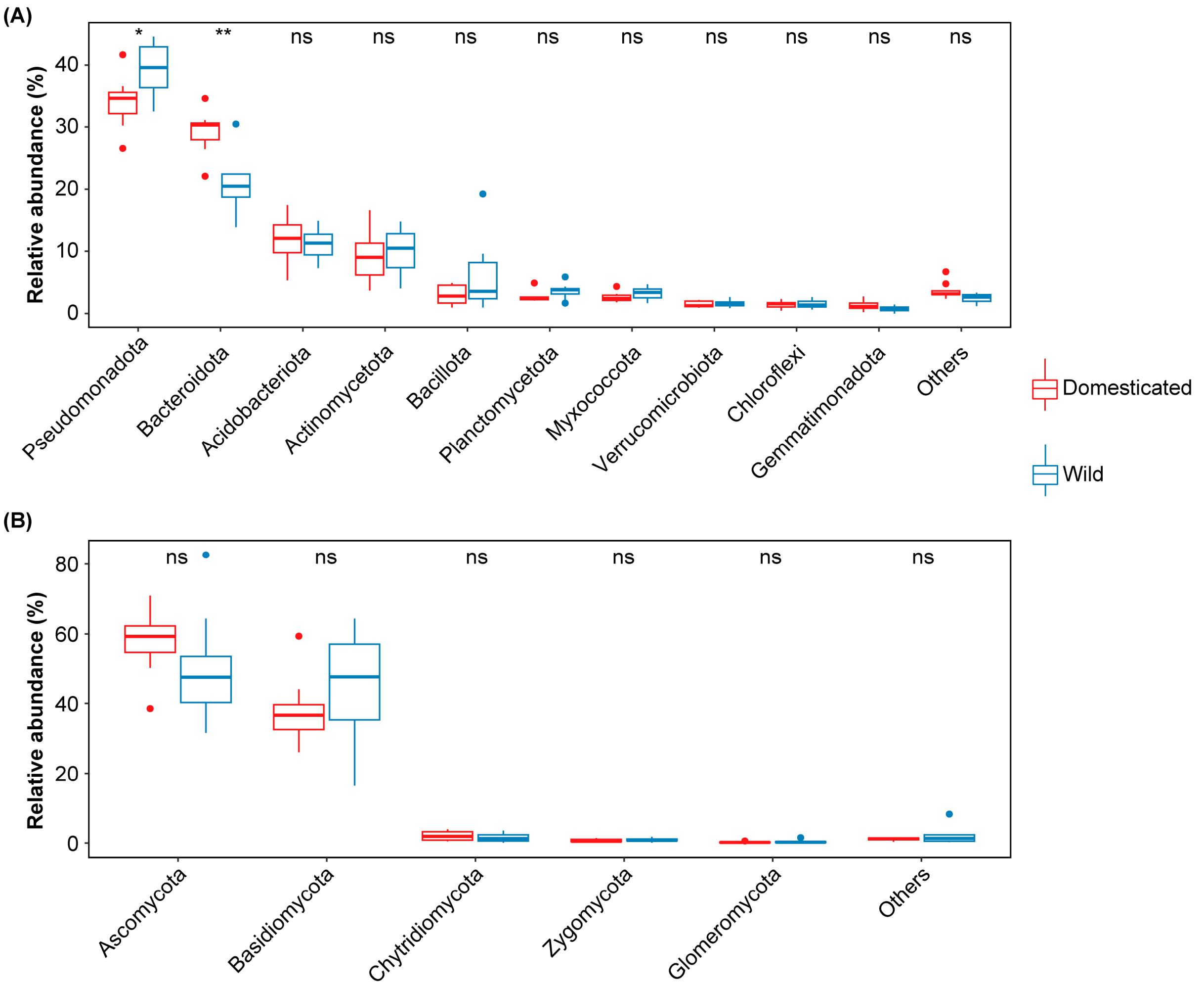

Supplementary Figure S2 Boxplots comparing the relative abundances of most abundant bacterial (A) and fungal (B) phylum in rhizosphere soils of the wild wheat and domesticated wheat. The abundant bacterial phyla with an average relative abundance of more than 1% are shown, while other, less abundant phyla and unclassified sequences are combined into “Others”. All the fungal phyla are shown, but for some unclassified sequences are combined into “Others”. Statistical differences were analyzed using a pairwise Wilcoxon test, *** $p < 0.001$, ** $p < 0.01$, * $p < 0.05$; ns, not significant

Supplementary Figure S3

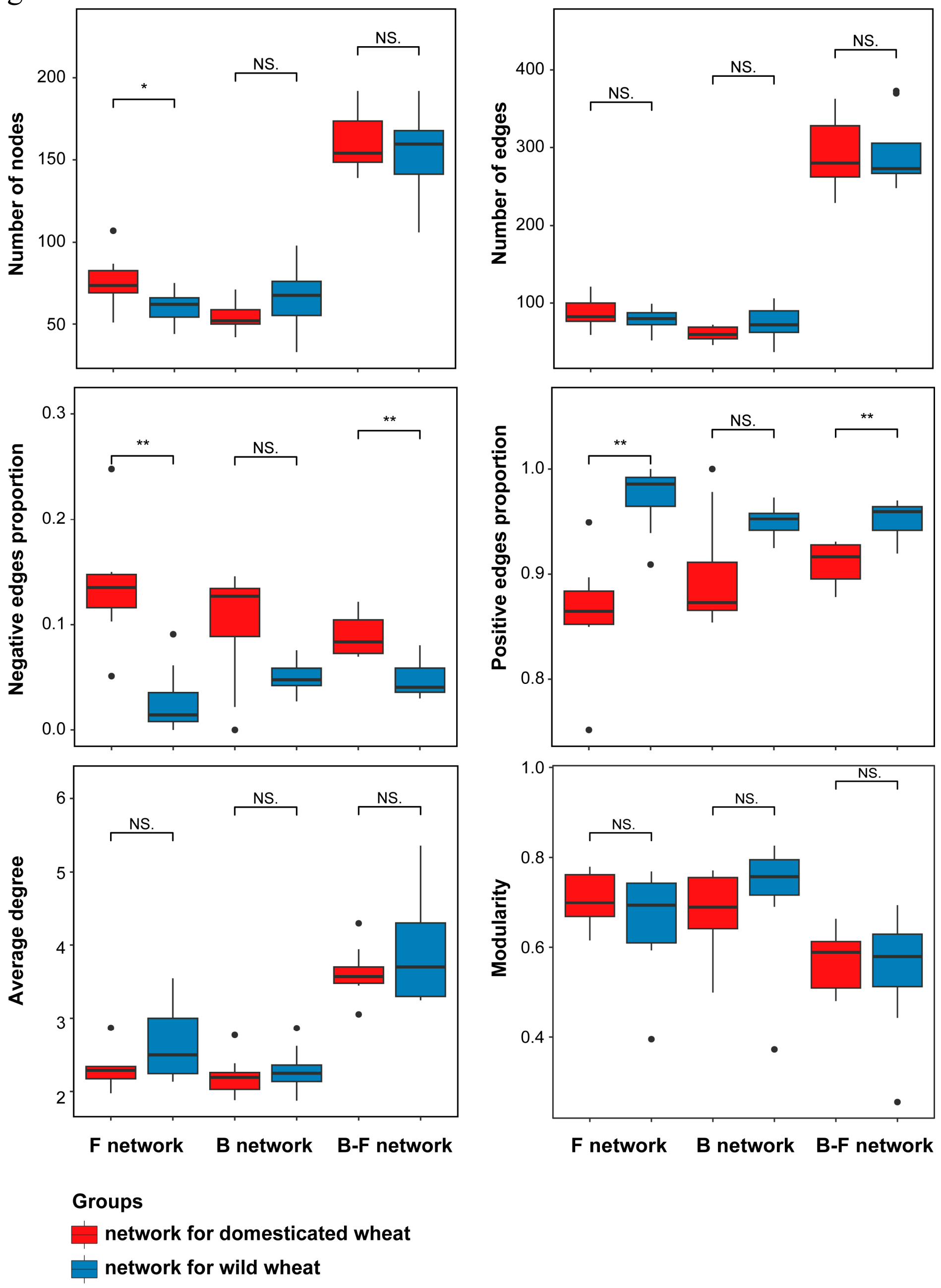

Supplementary Figure S3 Microbial network topologic feature comparison between domesticated and wild wheat. Statistical differences were analyzed using a pairwise Wilcoxon test, *** $p < 0.001$, ** $p < 0.01$, * $p < 0.05$; ns, not significant

Supplementary Figure S4

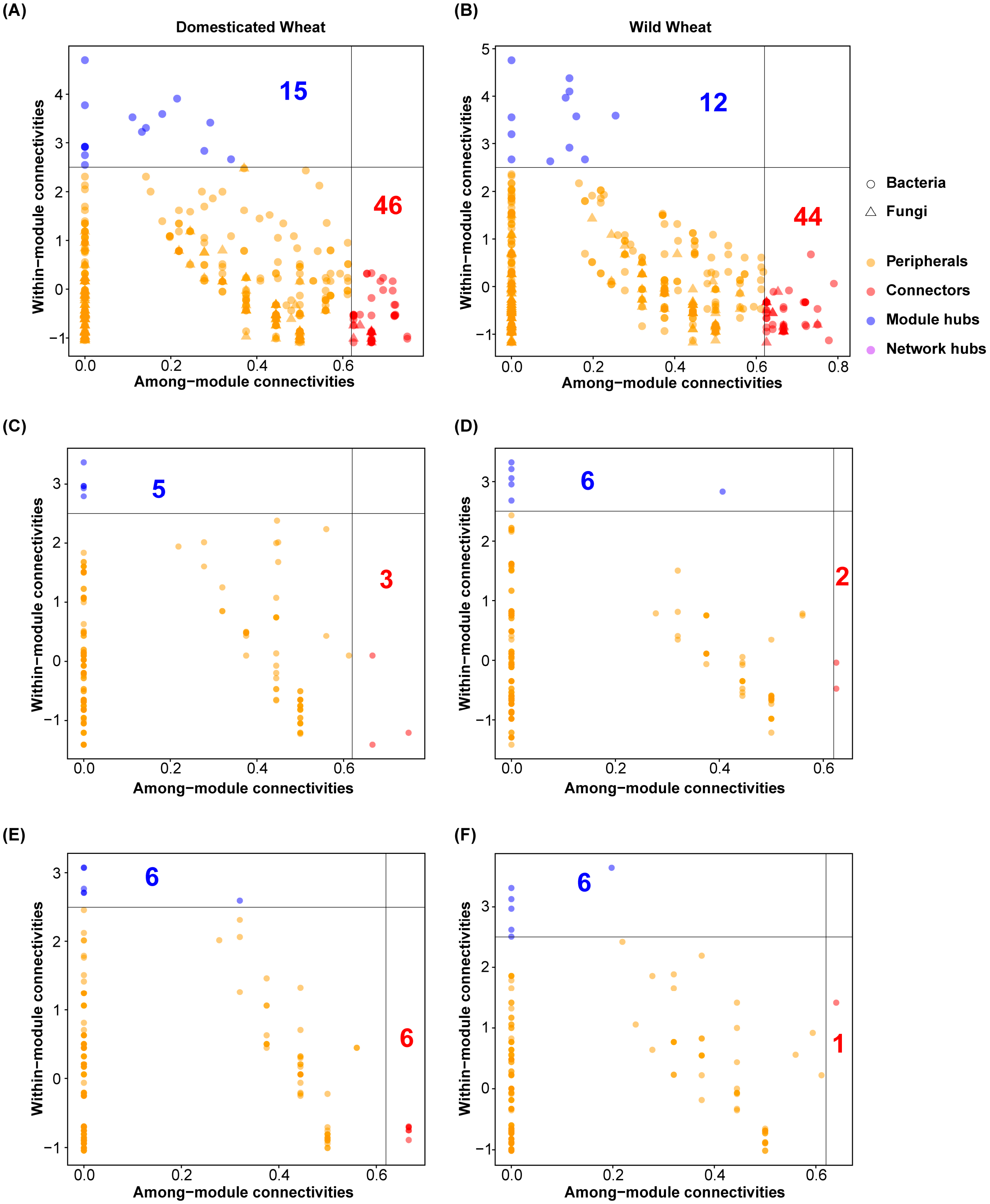

Supplementary Figure S4 Keystone species in microbial networks. Plots showed key taxa in (A and B) bacteria-fungi networks, in (C and D) bacteria single networks, and in (E and F) fungi single networks of domesticated and wild wheat rhizosphere soils, respectively.
